# Unveiling the genomic architecture of multidrug-resistant *Pseudomonas* sp. RTCS2 isolated from spoiled *Solanum lycopersicum* L.

**DOI:** 10.1128/mra.00337-25

**Published:** 2025-06-09

**Authors:** Shubhajit Shaw, Rittick Mondal, Pankaj Mandal, Avijit Mandal, Ritwik Acharya, Dipanjan Das, Sujan Ch. Paul, Arka Pratim Chakraborty, Debnirmalya Gangopadhyay, Amit Kumar Mandal

**Affiliations:** 1 Department of Sericulture, Raiganj University 561306, Raiganj, West Bengal, India; 2 Department of Life Sciences, Presidency University 568916, Kolkata, West Bengal, India; 3 Department of Botany, Raiganj University 561306, Raiganj, West Bengal, India; The University of Arizona, Tucson, Arizona, USA

**Keywords:** draft genome, tomato spoilage bacterium

## Abstract

Here, we report the draft genome of *Pseudomonas* sp. RTCS2, a gram-negative rod-shaped bacterium associated with tomato spoilage, isolated from Raiganj University, India (25.6071° N; 88.1306° E). The 5,526,005 bp genome with a 62.9% G + C content harbors antimicrobial resistance genes (*adeF*, *vanH*, and *vanT*), highlighting its pathogenic potential.

## ANNOUNCEMENT

**Fig 1:**
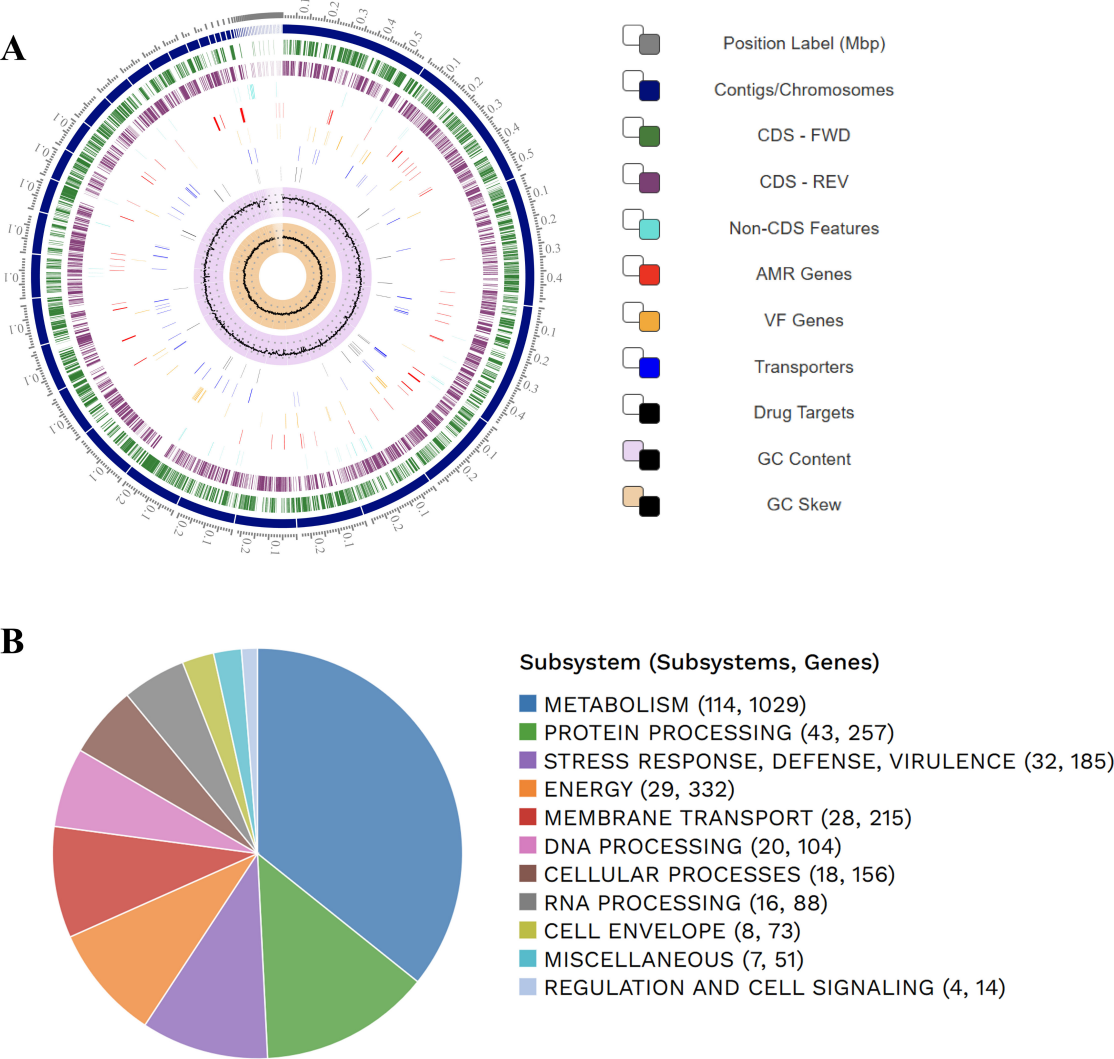
(**A**) Circular view of the RTCS2 genome showing from outer to inner rings, the contigs, CDS on the forward strand, CDS on the reverse strand, RNA genes, CDS with homology to known antimicrobial resistance genes, CDS with homology to known virulence factors, GC content, and GC skewness. (**B**) Subsystem analysis of the RTCS2 genome showing the metabolic profile.

Tomato (*Solanum lycopersicum* L.) suffers major losses from *Pseudomonas* spp., which rapidly degrade tomato due to its high water content (1–3). In our previous study on food preservation using silk sericin-based silver nanocomposites, *Pseudomonas* sp. strain RTCS2 was isolated from spoiled tomato surfaces. Taxonomic identification via 16S rRNA sequencing, following Shaw et al. (4), showed that RTCS2 is closely related to *Pseudomonas asiatica* RYU5 (Accession No. MH517510). The sample was collected using a sterile swab, diluted in PBS, and spread on a nutrient agar plate (composition: peptone, 5.0 g; sodium chloride, 5.0 g; HM peptone B, 1.5 g, yeast extract, 1.5 g, agar, 15.0 g, distilled water, 1 L, pH 7.4), followed by overnight incubation at 37°C. Thereafter, single colonies were isolated, sub-cultured, and maintained for 12 generations before preservation at −80°C in 20% glycerol in the Chemical Biology Laboratory, Raiganj University, with the internal reference code RTCS2. For genomic DNA extraction, a single colony was grown in nutrient broth at 37°C overnight, and biomass was pelleted by centrifugation, followed by extraction using the standard phenol-chloroform method (5). Paired-end libraries were then prepared and sequenced using the Illumina NovaSeq 6000 platform (Neuberg Diagnostics Pvt. Ltd., Ahmedabad, India). The DNA library was prepared using the KAPA HyperPlus Kit (Roche #07962428001). The final DNA libraries were quantified using the Qubit 4.0 fluorometer (ThermoFisher #Q33238) using the DNA HS assay kit (ThermoFisher #Q32851) following the manufacturer’s protocol. The insert size of the library was identified using the TapeStation 4150 system (Agilent) utilizing highly sensitive D1000 Screentapes (Agilent # 5067-5582) following the manufacturer’s protocol, yielding 22,956,170 reads with a 2 × 150 bp paired-end read length. Quality assessment of the raw fastq reads of the sample was performed using FastQC v.0.11.9 (default parameters) (6). The raw fastq reads were preprocessed using Fastp v.0.23.4 (7) (parameters: *--length_required 50—correction—trim_poly_g --qualified_quality_phred30—unqualified_percent_limit*30—average_qual 30). Processed data were re-assessed using FastQC. The read-level taxonomy profiling of the processed data was done using Kraken2 v.2.1.3 bacteria databases (8). The processed reads (Phred score >Q30) were *de novo-*assembled using Unicycler v.0.4.4 with default parameters (9). Estimations of the completeness and rate of contamination of the assembled genome were done using CheckM2 v.1.0.1 (10). The annotation was carried out via the NCBI Prokaryotic Genome Annotation Pipeline (PGAP) v6.9 with the methods best-placed reference protein set and GeneMarkS-2+ (11). The assembly produced a draft genome sequence encompassing 63 contigs. The *N*_50_ value is 246,858 bp, and the *L*_50_ count is 7. The estimated genome size is 5,526,005 bp, with a G + C content of 62.9% and 565.36 x genome coverage. A total of 5,048 coding sequences were annotated, including four rRNA genes (one 5S, one 16S, and two 23S rRNA genes) and 68 tRNA genes. Finally, a circular genome map and subsystem overview of RTCS2 was generated using BV-BRC v.3.47.8 (12) (Fig. 1). Resistance genes *adeF*, *vanH*, and *vanT* suggest RTCS2’s pathogenic potential, warranting surveillance and future functional validation.

## Data Availability

This whole-genome shotgun project data have been deposited at the NCBI under the accession number JBLKPS000000000. The version described in this paper is the first version, JBLKPS000000000.1. The BioSample and BioProject accession numbers are SAMN45968042 and PRJNA1203019, respectively. The raw data are available from the Sequence Read Archive (SRA) under the accession number SRX27219549.
